# Thiopurine Drugs in the Treatment of Ulcerative Colitis: Identification of a Novel Deleterious Mutation in TPMT

**DOI:** 10.3390/genes11101212

**Published:** 2020-10-16

**Authors:** Pierre-Olivier Harmand, Jérôme Solassol

**Affiliations:** 1 Laboratoire de Biologie des Tumeurs Solides, Département de Pathologie et Oncobiologie, Centre Hospitalier Universitaire de Montpellier, 34295 Montpellier, France; po-harmand@chu-montpellier.fr; 2 Institut de Recherche en Cancérologie de Montpellier, INSERM, Université de Montpellier, Institut Régional du Cancer de Montpellier, 34298 Montpellier, France

**Keywords:** TPMT, ulcerative colitis, azathioprine, mutation, targeted therapies

## Abstract

Chronic inflammatory bowel disease (IBD) includes Crohn’s disease and ulcerative colitis. Both are characterized by inflammation of part of the digestive tract lining. Azathioprine (AZA) is a well-known immunosuppressant that has been known for many years for its ability to provide long-term disease remission in IBDs, but has important side effects, most of which are related to a single nucleotide polymorphism in the gene for thiopurine methyltransferase (TPMT), which ensures the degradation and efficacy of AZA. Since a direct correlation between *TPMT* gene polymorphisms and the haematological toxicity of the AZA treatment has been widely demonstrated, *TPMT* genotyping has been made necessary prior to any introduction of AZA. The monitoring of thiopurine metabolites presents one of the factors that limit wide adaptation of these thiopurines in clinical practice. Thus, identifying patients with asymmetric metabolism could help clinicians provide an ideal treatment recommendation to improve response and reduce adverse effects. Here, we review the role of AZA in the treatment of IBD and discuss the usefulness of *TPMT* genotyping to guide clinical decision-making. In addition, we report the identification of a new molecular alteration, never described, *TPMT* mutation affecting the TPMT activity and responsible for deleterious side effects in a clinical case of a 20-year-old woman patient.

## 1. Introduction

Inflammatory bowel disease (IBD) is a term expressed to define a set of intestinal disorders involved in the whole (or part) of the digestive tract, comprising two main pathologies: Ulcerative colitis (UC) and Crohn’s disease (CD). IBD affects 0.3% of the population of Europe and North America, and tends to increase in populations of countries with increasing industrialization (Africa, South America, Asia, Middle East) [1,2]. Although UC and CD share important clinical signs, their symptoms differ greatly in their location and treatment to address them. Indeed, UC is located only in the inner wall of the large colon, while CD is involved in the entire digestive tract (from the esophagus to the rectum), but also in other organs such as the skin, liver, or eyes. The immune system of people with IBD does not respond well to environmental triggers, activating T-cells and triggering chronic inflammation of the gastrointestinal tract [3].

Aminosalicylated derivatives such as mesalazine or sulfasalazine are drugs prescribed in first line to induce and maintain remission in UC; to which if necessary, glucocorticoids can be added. Cyclosporine is used in more critical UC, if intravenous steroids do not produce the desired induction of remission. Azathioprine (AZA) and 6-mercaptopurine (6-MP), two analogues of thiopurine, have been the mainstay of IBD treatment as steroid sparing agents thanks to their modulation of the immune system [4,5]. They are used to maintain the remission acquired during the use of corticosteroids, but especially in combination with biological therapy. More recently, chimeric monoclonal antibodies anti-TNF-α (TNF-α) (infliximab and adalimumab) have been proposed for the induction of remission and the maintenance therapy of therapy-refractory UC [6].

The combination of immunosuppressants and biotherapies that have been shown to be effective has been reported to have better results, either by potentiating immunosuppression or by decreasing the immunogenicity inherent in biotherapy [7]. Thus, given the high risk of relapse in CD, the high dependence on corticosteroids, and the high response rate to the use of immunosuppressants when introduced early, azathioprine is the most appropriate drug to maintain the remission achieved through the use of systemic corticosteroids. However, there is considerable inter-patient variability which explains the clinically observed differences in the efficacy and toxicity of azathioprine and 6-mercaptopurine. Among the factors that may influence the response to pharmacotherapy, and the risk of developing significant and disabling adverse events (AEs) are environmental factors such as smoking but also, more importantly, genetic factors [8,9]. Thus, it is estimated that polymorphisms of certain genes can explain up to 95% of the observed variability in drug effects. Primary candidate genes of interest include those encoding drug receptors, metabolizing enzymes, and transporters. Therefore, since a direct correlation between TPMT gene polymorphisms and hematological toxicity of thiopurine treatment has been largely demonstrated, *TPMT* genotyping has been rendered necessary prior to any AZA introduction.

In this review, we summarize the main pharmacological features of AZA and 6-MP, describe the genetic variants in *TPMT* that influence their toxicity, and report the identification of a new molecular alteration responsible for deleterious side effects in a clinical case.

## 2. Azathioprine and 6-Mercaptopurine

6-MP or the prodrug AZA are purine analogues that stop the synthesis of adenine and guanine. This interruption causes a poor base incorporation and prevents the proper development of DNA repair mechanisms [10,11,12]. At low doses, AZA has a considerable effect on T-lymphocytes; it acts as an anti-inflammatory; whereas in high doses, it has an immunosuppressive and above all cytotoxic effect [3]. The molecules of the thiopurine family are conventionally used to treat malignant tumors, rheumatic diseases, and dermatological conditions; but they are also employed in the prevention of post-transplant organ rejection and in the treatment of inflammatory gastrointestinal disorders such as IBD.

Initially, in patients with CD, AZA was administered at a low dose (1.0–2.5 mg/kg/day) and was shown to be effective in reducing the risk of disease recurrence for a period ranging from 6 months to 2 years [13]. However, very quickly, the conventional AZA administration strategy was defined by a method of constant dose increase to avoid undesirable events, such as myelosuppression or hepatotoxicity [14]. 

Thiopurines are normally administered to sustain long-term remission because of their slow onset, requiring at least 12 to 17 weeks of regular treatment to produce discernible results. In order to more rapidly induce remission during the initial stages of IBD treatment, thiopurines can be combined with a short-term treatment: Typically, a steroid or anti-TNF agent. It is not wholly understood how the combination of immunosuppressants and anti-TNF agents operates, but any beneficial synergy could be due to the improved pharmacokinetic profiles produced by the interaction of the drugs combined with the presence of multiple therapeutic targets. The combination of anti-TNF therapies with thiopurines can induce better therapeutic efficacy [15,16]. The implication of AZA monotherapy in IBD for the maintenance of remission is still a matter of debate for clinicians. However, the European organization of IBD has published a consensus statement in which clinicians agree to the use of thiopurines as monotherapy or in addition to infliximab against corticosteroid-dependent IBDs, stating the significant efficacy of thiopurines compared with aminosalicylates to slow the rise of UC [17,18].

### 2.1. Mechanism of Action 

**Figure 1:**
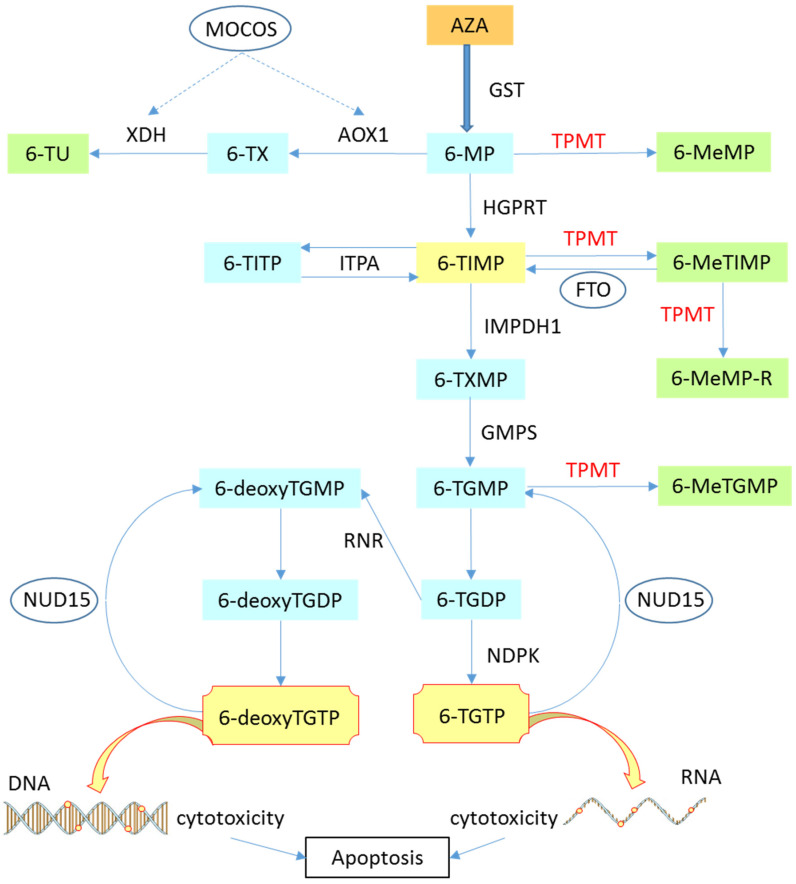
Involvement of TPMT on the thiopurine metabolic pathway. AZA (azathioprine); AOX1 (aldehyde oxidase type 1); FTO (fat mass and obesity associated); GST (gluthation s-transferase); GMPS (guanosine monophosphate synthetase); HGPRT (hypoxanthine guanine phosphoribosyl transferase); IMPDH1 (inosine 5-monophosphate deshydrogenase type 1); IPTA (inosine triphosphate pyrophosphatase); MOCOS (molybdenum cofactor sulfurase); NDPK (nucleotide diphosphate kinase); NUD15 (nucleoside diphosphate linked moiety X type 15); RNR (ribonucleotide reductase); TPMT (thiopurine s-methyltransferase); XDH (synonym-Xanthine oxidase); 6-MP (6-mercaptopurine); 6-MeMP (6-methyl-mercaptopurine); 6-MeMPR (6-methyl-mercaptopurine ribonucleotide); 6-MeTGMP (6-methyl-thioguanine monophosphate); 6-MeTIMP (6-methyl-thioinosine monophosphate); 6-deoxyTGMP (6-deoxy-thioguanine monophosphate); 6-deoxyTGDP (6-deoxy-thioguanine diphosphate); 6-deoxyTGTP (6-deoxy-thioguanine triphosphate); 6-TGMP (6- thioguanine monophosphate); 6-TGDP (6-thioguanine diphosphate); 6-TGTP (6-thioguanine triphosphate); 6-TIMP (6-thioinosine monophosphate); 6-TITP (6-thionosine triphosphate); 6-TXMP (6-thioxanthine monophsphate); 6-TU (thiouric acid); 6-TX (6-thioxanthine).

AZA plays an important role in the treatment of autoimmune diseases. It is a prodrug converted to 6-MP in vivo where it is then metabolized into the active metabolite 6-thioguanine triphosphate (6-TGTP) by a cascade of enzymes (hypoxanthine guanine phosphoribosyl transferase (HGPRT), inosine monophosphate dehydrogenase (IMPDH), guanosine monophosphate synthetase (GMPS), and nucleotide diphosphate kinase (DNPK)) or in inactive metabolites as 6-methylmercaptopurine (6-MeMP), 6-methylmercaptopurine ribonucleotide (6-MeMPR), 6-methyl thioinosine monophosphate MeTIMP), or as 6-methylthioguanosine monophosphate (6-MeTGMP) by the enzyme TPMT [19,20]. Thus, the 6-thioguanosine nucleotides (6-TGN = 6-TGTP) determines the cytotoxicity or efficacy of AZA. As shown in Figure 1, there are three metabolic pathways of AZA: Once in the form of 6-MP, the latter can be converted into thiouric acid (6-TU) through the AOX1 and XDH enzymes of the family Xanthine oxidases (XO). 6-MP can also be converted to 6-TIMP (6-thioinosine monophosphate) by the enzyme HGPRT, which will then be converted to active metabolites such as 6-TGTP and 6-deoxyTGTP by IMPDH1, GMPS, NDPK, and ribonucloside reductase (RNR) (for 6-deoxyTGTP). Then, 6-TGTP and 6-deoxyTGTP obtained, respectively induce cytotoxicity on RNA and DNA by poor incorporation of nucleotides. Finally, thiopurine-S-methyltranferase (TPMT) will induce the methylation of each metabolite of AZA (6-MP to 6-MeMP, 6-TIMP to 6-MeTIMP, and 6-TGMP to 6-MeTGMP) in order to make them inactive, and thus prevent the cytotoxic function of the AZA metabolic pathway [21]. In order to determine the amount of 6-MP catalyzed into 6-TGN, the TPMT enzyme directly competes with HGPRT and XO [22,23].

The metabolic pathway for AZA is developing, but is not yet well defined. Genes such as MOCOS, NUD15, and FTO are thought to be involved in the metabolism of AZA [24,25,26,27]. The MOCOS gene (molybdenum cofactor sulfurase) is thought to have an action on the enzymes AOX1 and XDH [25,28], while the NUD15 gene (nucleoside diphosphate linked moiety X type 15) dephosphoryl 6-TGTP into 6-TGMP [27,29]. As for the FTO gene (fat mass and obesity associated), it could be a potential pharmacogenetic biomarker for treatment with thiopurine [24]. In order to better define the roles of these genes, studies are currently underway.

At normal levels of TPMT activity, 6-TGTP and 6-deoxyTGTP inhibit intracellular signaling pathways and induce lymphocytic apoptosis. A change in these activity levels necessarily creates an imbalance: In fact, an abnormal increase in the TPMT enzyme activity induces a decrease in 6-TGN and therefore a decrease in the effectiveness of the drug. Conversely, the decrease in the TPMT activity, or even a total absence of TPMT activity (as observed when TPMT exhibits polymorphisms) this time leads to a significant increase in the presence of 6-TGN, which will be incorporated into DNA and trigger cytotoxicity [30,31].

### 2.2. Pharmacology 

Currently, AZA is administered intravenously (current dose of 1 to 1.5 mg/kg) or orally (daily dose of 0.5 mg/kg), either as a tablet or an oral delayed release (DR) capsule. In 1996, Van Os et al. [32] carried out a comparative study on the different types of administration of AZA (50 mg): Intravenous, oral tablet, oral DR capsule, and hydrophilic or hydrophobic rectal foam. Rectal foam has been found to be ideal because its application to the colon avoids first-pass metabolism by the liver. In contrast, it is a low-intensity process because its absorption through the colon mucosa is reduced compared to that in the gastric [32]. In erythrocytes, the half-life of AZA would be 25 to 80 min (or 3 to 5 h if the metabolites are included) [33], while that of 6-TGN would be 5 days, inducing a period long enough of 3 to 6 months necessary to reach a state of equilibrium [34]. This may explain why an extended treatment period is necessary before a clinical response occurs.

In clinical practice, AZA is systematically administered initially at a low dose (25 to 50 mg/day); then the dose is increased by 25 to 50 mg/day every 1 to 2 weeks, up to 2.0 to 2.5 mg/kg, with regular monitoring in order to detect the appearance of AEs, in particular haematological or hepatic [35,36]. Thus, the first phase therapeutic strategy based on the remission induction treatment is followed by the second phase to maintain remission. However, this progressive titration method is still insufficient to anticipate the onset of severe myelosuppression. The dose of 6-mercaptopurine in mg/kg is equivalent to a factor of 2.08 times the pharmaceutical dose of AZA [37].

### 2.3. Thiopurine Side Effects

Many side effects induced by AZA have been reported including nausea, myelosuppression, erythrocyte aplasia, and in very rare cases lead to death [38]. All of these effects have been categorized into two groups of reactions: One with dose-independent reactions and the other dose-dependent. The reactions induced by AZA given initially in the first few weeks tend to be hypersensitive reactions of the allergic type. Dose-independent reactions cause symptoms such as fever, joint pain, rash, pancreatitis, or gastrointestinal disturbances [39]. While dose-dependent reactions appear at post-treatment stages due to accumulated metabolites and are often characterized by symptoms such as rare bacterial infections, nausea, leukopenia, hepatitis, cholestatic jaundice, and myelosuppression. If the side effects associated with the dose-dependent disappear as soon as the dose of AZA is lowered, the dose-independent reactions continue until the treatment is stopped.

Many studies disagree on the significant association between the thiopurine treatment for patients with IBD and the increased risk of malignant tumors [40,41,42]. According to Lewis et al., thiopurine remains a major molecule in the treatment against IBD, despite a risk factor for lymphoma multiplied by 4. The increased risk would have to be greater than 9.8-fold for the benefit of AZA therapy to be replaced by the benefit of alternative therapies [34,43]. Usually, patients treated with AZA will also receive combination therapy with a corticosteroid, the dose of which will be gradually reduced until it is stopped once the remission status of the condition is reached. Indeed, the study by De Jong et al. shows that patients receiving this combined therapy, with a higher dose of corticosteroid, have a lower number of adverse events and especially less serious [44]. In addition, it has been determined that 20–30% of patients treated with AZA monotherapy alone discontinue treatment due to an adverse drug reaction [8,23].

In order to determine an individual’s risk of major toxicity, the level of TPMT activity is measured. This process makes it possible to adjust the dose or even avoid the administration of the drug, and thus prevents the risk for the patient of developing myelosuppression, neutropenia, leukopenia, or thrombocytopenia. Thus, determination of the concentrations of 6-TGN and 6-MP in red blood cells after at least four weeks of therapy has been proposed and a level of 235–450 pmol/8 × 108 erythrocytes of 6-TGN has been recommended [27,45]. It is estimated that 20% of treated patients exhibit hypermethylation of AZA, inducing an increase in 6-MMp with low levels of 6-TGN. The metabolism of AZA is skewed, resulting in treatment failure [46]. These elevated levels of 6-MMP tend to increase hepatotoxicity, causing the 6-MMP/6-TGN ratio to increase; which results in a low therapeutic efficacy [47]. Thus, the identification of patients with asymmetric metabolism would be a major asset for improving the therapeutic response and above all reducing adverse events [29,48].

### 2.4. Pharmagenetics

Genetic variations of genes encoding enzymes involved in thiopurine metabolism give rise to varying degrees of therapeutic response and toxicity. Indeed, these allelic variants are known as single-nucleotide polymorphisms (SNPs), which are characterized by a substitution of one base for another in the human DNA sequence. A SNP is a genetic mutation whose specific allele switch is observed in more than 1% of the population [49]. We can see in Figure 1, that TPMT, HGPRT, and xanthine oxidase are the enzymes involved in the metabolism of AZA. The formation of SNPs on these enzymes result in variable active metabolites that influence the metabolism of AZA. The TPMT gene is the most studied [50].

The genetic polymorphisms of *TPMT* have been well-documented as causing myelosuppression when patients are treated with azathioprine. This is due to the inability of the TPMT enzyme to metabolize 6-MP, resulting in the conversion of 6-TGN and, when incorporated into DNA, exerts cytotoxicity which triggers the arrest of the cell cycle and apoptosis [51]. Therefore, since *TPMT* polymorphisms have been associated with many severe thiopurine-induced adverse drug reactions, prior to starting thiopurine therapy, genotyping of common *TPMT* alleles is now recommended in the clinical practice. To precisely define those at risk and optimize therapy, the identification of new variants is essential. 

## 3. TPMT

The *TPMT* gene located on chromosome 6 (6p22.3) is 34 kb long and consists of 10 exons, eight of which encode the protein thiopurine methyltransferase (28 kDa) [11]. Thiopurine S-methyltransferase is a cytosolic enzyme which catalyzes the S-methylation of aromatic or heterocyclic sulfhydryl compounds, especially exogenous substances such as 6-MP. The *TPMT* gene exhibits an autosomal codominant genetic polymorphism, which can lead to an absence or a low level of TPMT activity in heterozygous or homozygous individuals [22]. 

The presence of *TPMT* polymorphisms should be considered and especially not overlooked in the treatment with thiopurine-based drugs, as they may affect the tolerance and efficacy of the drugs. In fact, the activity of *TPMT* in humans is extremely variable from one individual to another and divides the population into three categories: A majority group of subjects for whom the TPMT activity is high (or normal), a group of individuals for whom the activity is said to be intermediate, and a small group of subjects for whom the TPMT activity is undetectable [51,52]. 

The TPMT activity phenotype is inherited in the autosomal co-dominant mode; thus, the deficient individuals are homozygous or heterozygous for one or two non-functional alleles of the *TPMT* gene, while the intermediate individuals are heterozygous, that is to say carriers of a functional allele and a non-functional allele of the gene. Patients with intermediate TPMT activity have a high probability of reporting adverse events if treated with standard doses of thiopurines. Conversely, these same patients may not have major side effects when the dose is properly adjusted [53]. 

The hereditary nature of TPMT activity deficit has been shown in family studies. In fact, in the Caucasian population of Europe and North America, approximately 89% of subjects have a high (or normal) enzyme activity, 11% of them have an intermediate activity, while less than 1% have a total deficit of activity [54]. 

Analysis of the TPMT gene sequence, performed in populations of various ethnic origins, has confirmed the genetic origin of the TPMT activity polymorphism and elucidated its molecular mechanisms [55], and identified more than forty allelic variants of the *TPMT* gene [56].

**Figure 2:**
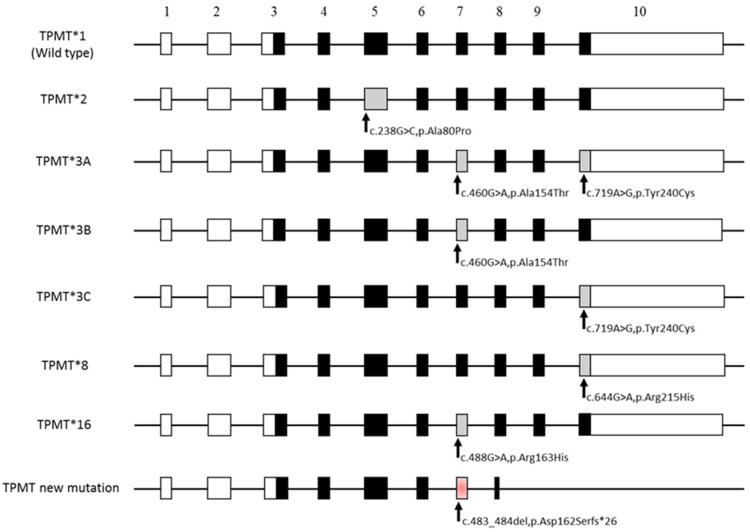
Allelic variants of the human TPMT locus. The TPMT gene is located on 6p22.3 (NM_000367.2, OMIM 187680) and comprises 10 exons and nine introns. Boxes depict exons in the human *TPMT* gene and spaces between boxes are introns. White boxes show untranslated exonic regions and black boxes represent exons in the open reading frame (ORF). Grey boxes correspond to different exons with molecular alterations inducing changes in amino acids. The positions of the most frequent SNPs are indicated by vertical arrows. The red box shows exon 7 with the c.483_484del, p.Asp162Serfs * 26 mutation inducing a TPMT * 16 mutation (c.488G > A,p.Arg163His).

The wild type TPMT * 1/TPMT * 1 genotype is consistent with the normal TPMT enzymatic activity. Standard doses of thiopurine drugs are less likely to be toxic in individuals with this genotype. It is retained that the heterozygotes with 1 wild-type allele and 1 variant (such as the TPMT * 1/* 2, TPMT * 1/TPMT * 1/* 3A, TPMT * 1/* 3B, TPMT * 1/* 3C, TPMT genotypes * 1/* 8 and TPMT * 1/* 16) have an intermediate TPMT activity, have an increased risk of haematological toxicity, and may require a lower dosage (Figure 2). The star alleles described are referenced in the TPMT nomenclature committee database (https://liu.se/en/research/tpmt-nomenclature-committee) which lists the TPMT variants. The Pharmacogenic Variation Consortium (PharmVar) continues to update new alleles to provide the clinical and research communities of a repository and standardized nomenclature of pharmacogene variation.

Patients who lack the wild-type allele are predicted to have low or no detectable enzyme activity and are at high risk for life-threatening haematologic toxicity if given full doses of thiopurine medication. Alternative therapy or reduced dosage should be considered for these patients.

It should be noted that the TPMT * 3B, TPMT * 3C, TPMT * 8, and TPMT * 16 alleles each carry a variant which is specific to it. However, some alleles carry two variants such as the TPMT * 3A allele, which contains the polymorphisms found in the TPMT * 3B and TPMT * 3C alleles.

TPMT * 2, * 3A, and * 3C represent more than 90% of inactive alleles [57]. Among the most common is the TPMT * 2 allele, which compared to the wild-type sequence of the gene (TPMT * 1), carries a missense mutation in exon 5 (c.238G > C, p.Ala80Pro). This sequence variation alters the tertiary structure of the protein, which alters the stability of the enzyme, and leads to its rapid degradation by proteolysis [58]. The TPMT * 3A allele harbors two missense variants in Cis found in the TPMT * 3B (c.460G > A, p.Ala154Thr) and TPMT * 3C (c.719A > G, p.Tyr240Cys) alleles, located at exons 7 and 10, respectively. These two-point mutations, present in most Caucasians with a phenotype with undetectable TPMT activity, lead to a decrease in the quantity of protein [59]. This reduction would be the result of a post-translational mechanism linked to an activation of the proteolytic pathways, the consequence of which is the increase in the rate of degradation of the enzyme by the proteasome [58,60]. Finally, the third non-functional allelic variant, the TPMT * 3B allele, contains only the c.460G > A, p.Ala154Thr mutation in exon 7 [59].

These three mutations, c.238G > C,p.Ala80Pro, c.460G > A,p.Ala154Thr, and c.719A > G,p.Tyr240Cys, are at the origin of the non-functional alleles of *TPMT* most frequently encountered regardless of the ethnic origin of individuals, with increasing frequency c.238G > C, c.460G > A, c.719A > G [61] (see Table 1). 

## 4. Case Report

A 20-year-old woman was diagnosed with UC based on standard clinical, endoscopic, and histological criteria. The patient began to take 4 g of mesalazine per day but heart palpitations rapidly (tachychardia) occurred and the treatment was stopped. Subsequently, this young woman was treated with prednisolone and 6-mercaptopurine, but therapy was also stopped due to leukopenia. *TPMT* genotyping was performed by sequence analysis of all coding exons and flanking intron regions of the *TPMT* gene and the result was available after the patient received azathioprine. Known “hot spot” mutations (Table 2) on exons 5, 7, and 10 of the *TPMT* gene were carried out by Sanger sequencing. 

**Figure 3:**
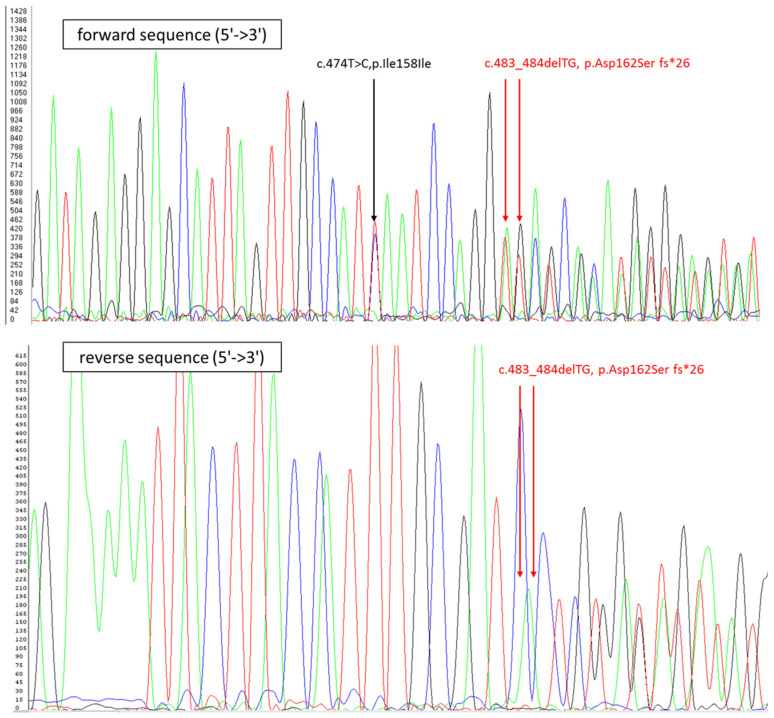
Electropherogram obtained with the SeqScape v2.5v software ( APPLIED Biosystem, Foster City, CA, USA) representing part of the sequence of TPMT exon 7. The black arrow shows a silencing mutation. The red arrow shows a novel c.483_484del, p.Asp162Serfs * 26 mutation that generates a premature stop codon in exon 8.

The genotyping of TPMT * 2, * 3A, and * 3C mutations were not detected. However, a novel class 5 deleterious mutation, c.483_484del, p.Asp162Serfs * 26 (submitted to the TPMT nomenclature committee), was found in exon 7 (Figure 3). This deletion involves a shift in the reading frame of the sequence and induces a STOP codon located at position 188 of the amino acids (Tyrosine → Stop) in the middle of exon 8. This molecular alteration generates a truncated TPMT protein without exons 9 and 10. In addition, the TPMT * 16 “hot spot” appears (G488A), inducing a mutation in one of the alleles. 

Briefly, the PCR reaction was carried out in a reaction mixture of 50 μL containing nuclease free water, 10 × buffer, 25 mM MgCl2, 5 mM of each deoxyribonucleotide triphosphate, 10 µM of each primer (sense primer 5′-CTCCACACCCAGGTCCACACATT-3 ’and reverse primer 5′-GTATAGTATACTAAAAAATTAAGACAGCTAAAC-3′), 5 U/µL of HotStar Taq DNA polymerase (Qiagen, Hilden, GERMANY), and 100 ng of genomic DNA. Cycling conditions were an initial denaturation of 95 °C for 10 min, followed by 14 cycles of amplification at 95 °C for 40 s, 55 °C for 40 s, 72 °C for 50 s, followed by 21 cycles of amplification at 95 °C for 40 s, 60 °C for 40 s, 72 °C for 50 s, and a final extension step of 72 °C for 5 min. Ten microliters of PCR product was visualized on an ethidium bromide-stained 2% agarose gel. The remaining 10 µL of PCR product was purified using a filter plate Multiscreen^®^ (Merck, Millipore Ltd., Burlington, MA, USA) and sequenced in both directions using the ABI BigDye Terminator version 1.1 chemistry on an ABI 3130X l DNA Analyzer (Applied Biosystems, Foster City, CA, USA). 

Thereafter, the patient underwent a first line of anti–TNF-α biotherapies with adalimumab (160 mg, 80 mg, and 40 mg every 2 weeks), but after a transient improvement, the symptomatology recurred. She received vedolizumab and then therapeutic exhaustion occurred despite optimization with 300 mg monthly. Then, golimumab (100 mg) was administered monthly with a partial improvement. Finally, she received ustekinumab with initial improvement but a recurrence 3 months later in the form of severe acute colic with a 6 kg weight loss stopped this therapy. As the disease had not been brought under control, treatment with azathioprine was recently reintroduced, but at a reduced dose because of a mutation in the *TPMT* gene. Finally, despite a combination therapy with optimized infleximab and AZA, the patient experienced disease progression and she initiated a new therapy with tofacitinib.

## 5. Conclusions

Although the role of azathioprine monotherapy in IBD for the maintenance of remission is still a point of discussion among clinicians, AZA remains an affordable and viable option for the public sector. However, genetic screening for the presence of SNP on the TPMT gene of patients with IBD is necessary to ensure therapeutic efficacy [62]. The mutations in the *TPMT* gene cause a TPMT deficiency, which is a reduction in the activity of the TPMT enzyme. Without enough of this enzyme, the body cannot “turn off” thiopurine drugs by metabolizing them into inactive compounds, damaging the bone marrow through haematopoietic toxicity. More than 20% of patients with inflammatory bowel disease stop the treatment due to serious side effects of the thiopurines [8,23]. Therefore, there are strong reasons to recommend that all patients be screened for TPMT deficiency before starting a course of these drugs. Measuring the enzyme activity remains a standard but this test is unfortunately unavailable in many centers. If necessary, low doses of azathioprine can be administered, which can be increased if the drug is tolerated. However, it should be noted that profound myelosuppression in TPMT-deficient patients is not always avoidable and the majority of cases of myelosuppression associated with thiopurines appear in individuals who fall into the normal range of TPMT activity. 

Genotyping can provide a useful adjunct in these situations. Here, we have reported a new clinically important TPMT * 16 mutation in a young patient who received the thiopurine treatment. This molecular alteration has never been described to our knowledge and is not referenced in genetic databases such as ClinVar, Alamut, or Ensembl. This reinforces the clinical significance of TPMT * 16 in the azathioprine treatment and also the utility of careful therapeutic monitoring of thiopurine metabolites in the presence of *TPMT* variants. With the reduction in the cost of DNA sequencing and an increase in sequence-based genotyping for non-synonymous SNPs using next-generation sequencing (NGS), an increasing number of new *TPMT* alleles are likely to be identified in the future.

It is important not to overlook the possibility of new genes involved in the thiopurine pathway. Indeed, a significant association has been identified between the enzymatic activity of TPMT and the MOCOS gene (molybdenum cofactor sulfurase). The latter is thought to have an action on the enzymes xanthine dehydrogenase (XDH) and aldeyde oxidase 1 (AOX1) involved in the degradation of thiopurines [63]. Moreover, the work of Coelho et al. [25] made it possible to detect a nominal association between tolerance to thiopurines and the GMPS gene (guanosine monophosphate synthetase). The involvement of this gene in the phosphorylation of 6-TIMP (6-thioinosine monophosphate) to 6-TGN (thioguanine nucleotides) allows thiopurines to exert their cytotoxic effects. Therefore, mutations in these MOCOS and GMPS genes would induce thiopurine toxicity. As the study by Coehlo et al. [25] points out, although NGS does not clearly provide an advantage as a biochemical test in predicting toxicity, it is a solid tool in the identification of pathogenic variants in patients not detected by the standard genotyping. The identification and then the study in NGS of a panel of genes involved in the metabolic pathway of thiopurine must be extended, in order to assess their toxicity and guide clinicians in the treatment strategy.

Thus, genotyping may become increasingly important in clinical practice because it provides clear benefits to the patient and allows the adaptation of pre-therapeutic doses for patients carrying the mutation.

## Figures and Tables

**Table 1:** Nomenclature of the most frequent non-functional alleles of the *TPMT* gene.

Common Allele Name	Exon	Coding DNA	Protein
TPMT * 2	5	c.238G > C	p.Ala80Pro
TPMT * 3A	7	c.460G > A	p.Ala154Thr
TPMT * 3A	10	c.719A > G	p.Tyr240Cys
TPMT * 3B	7	c.460G > A	p.Ala154Thr
TPMT * 3C	10	c.719A > G	p.Tyr240Cys
TPMT * 8	10	c.644G > A	p.Arg215His

**Table 2:** Genotyping of the *TPMT* gene-list of targeted molecular alterations.

EXON 5 :			EXON 7 :			EXON 10 :		
Variant	Nucleotide	Protein	Variant	Nucleotide	Protein	Variant	Nucleotide	Protein
TPMT * 2	c.238G > C	p.Ala80Pro	TPMT * 10	c.430G > C	p.Gly144Gln	TPMT * 8	c.644G > A	p.Arg215His
TPMT * 3D	c.292G > T	p.Glu98*	TPMT * 3A	c.460G > A	p.Ala154Arg	TPMT * 7	c.681T > G	p.His227Gln
TPMT * 9	c.356A > C	p.Lys119Thr	TPMT * 3B	c.460G > A	p.Ala154Arg	TPMT * 20	c.712A > G	p.Lys238Glu
TPMT * 19	c.365A > C	p.Lys122Thr	TPMT * 22	c.474T > C	p.Ile158Ile	TPMT * 3A	c.719A > G	p.Tyr240Cys
TPMT * 34	c.319T > G	p.Tyr107Asp	TPMT * 16	c.488G > A	p.Arg163His	TPMT * 3C	c.719A > G	p.tyr240Cys
TPMT * 28	c.349G > C	p.Gly117Arg	TPMT * 22	c.488G > C	p.Arg158Pro	TPMT * 41	c.719A > C	p.Tyr240Ser
TPMT * 32	c.340G > A	p.Glu114Lys	TPMT * 33	c.487C > T	p.Arg158Cys	TPMT * 25	c.634T > C	p.Cys212Ser
						TPMT * 37	c.648T > A	p.Cys216*
						TPMT * 40	c.677G > A	p.Arg226Gln
